# Acute effects of strength exercises and effects of regular strength training on cell free DNA concentrations in blood plasma

**DOI:** 10.1371/journal.pone.0184668

**Published:** 2017-09-14

**Authors:** Suzan Tug, Anna-Katharina Tross, Patrick Hegen, Elmo Wanja Immanuel Neuberger, Susanne Helmig, Wolfgang Schöllhorn, Perikles Simon

**Affiliations:** 1 Department of Sports Medicine, Disease Prevention and Rehabilitation, Johannes Gutenberg-University Mainz, Mainz, Germany; 2 Institute for Training and Movement Science, Johannes Gutenberg-University Mainz, Mainz, Germany; West Virginia University School of Medicine, UNITED STATES

## Abstract

Creatine kinase (CK) is a marker for muscle cell damage with limited potential as marker for training load in strength training. Recent exercise studies identified cell free DNA (cfDNA) as a marker for aseptic inflammation and cell damage. Here we overserved in a pilot study the acute effects during strength exercise and chronic effects of regular strength training on cfDNA concentrations over a period of four weeks in three training groups applying conservation training (CT) at 60% of the 1 repetition maximum, high intensity-low repetition training (HT) at 90% of the 1 repetition maximum and differential training (DT) at 60% of the 1 repetition maximum. EDTA-plasma samples were collected before every training session, and on the first and last training day repeatedly after every set of exercises. CfDNA increased significantly by 1.62-fold (mean (±SD) before first exercise: 8.31 (2.84) ng/ml, after last exercise 13.48 (4.12) ng/ml) across all groups within a single training session (p<0.001). The increase was 1.77-fold higher (mean (±SD) before first exercise: 12.23 (6.29) ng/ml, after last exercise 17.73 (11.24) ng/ml) in HT compared to CT (mean (±SD) before first exercise: 6.79 (1.28) ng/ml, after last exercise 10.05 (2.89) ng/ml) (p = 0.01). DNA size analysis suggested predominant release of short, mononucleosomal DNA-fragments in the acute exercise setting, while we detected an increase of mostly longer, polynucleosomal cfDNA-fragments at rest before the training session only at day two with a subsequent return to baseline (p<0.001). In contrast, training procedures did not cause any alterations in CK. Our results suggest that during strength exercise short-fragmented cfDNA is released, reflecting a fast, aseptic inflammatory response, while elevation of longer fragments at baseline on day two seemed to reflect mild cellular damage due to a novel training regime. We critically discuss the implications of our findings for future evaluations of cfDNA as a marker for training load in strength training.

## Introduction

The role of circulating cell free DNA (cfDNA) as a biological marker in blood plasma has gained more interest in various biomedical disciplines and exercise physiology [1–5]. Whereas the role of cfDNA under pathological conditions like cancer [6], sepsis [7] or stroke [8] has been intensively studied, there is still little knowledge about the influence of acute exercise bouts and chronic exercise regimens on cfDNA concentration. Levels of cfDNA have already been investigated in half- and ultra-marathons [3], weightlifting [9], long-term treadmill running [10], incremental treadmill tests [1,11], rowing exercises [5] and intensive cycling exercises [2,12] with a pronounced increase in cfDNA directly after cessation of exercise. In most studies cfDNA levels returned to baseline levels within two hours of recovery [1–5,9–11] and only after an ultra-marathon cfDNA levels remained elevated for up to 24h [3].

Currently, only one study analysed the acute effect of heavy weight lifting on cfDNA levels [9]. A single bout of high-intensive strength training led to an increase of cfDNA levels and caused more inflammation and/or muscle damage than aerobic exercise [9]. In a further study, several weeks of high intensity resistance training led to chronic elevations of baseline cfDNA concentrations which suggests that cfDNA could be used as a sensitive marker for exercise-induced cellular damage and inflammation following resistance training and for the diagnosis of overtraining [13]. Therefore, cfDNA may not only serve as a marker for disease-related conditions, but might also bear potential as an exercise biomarker. The advantage of cfDNA compared to creatine kinase could be a faster response following exercise, since elevations of cfDNA levels occur within minutes. In addition, cfDNA concentrations can be measured less invasively from capillary blood samples collected from the fingertip compared to venous blood samples [14]. In addition we can differentiate between mononucleosomal and polynucleosomal cfDNA levels using a direct qPCR, established as a simple, economic and sensitive procedure for the quantification of cfDNA concentrations from plasma [14]. Up to date, no study analysed the impact of strength training on the concentrations of cfDNA-fragment sizes in capillary blood during exercise and at rest over the course of a training session.

Therefore, the aim of our pilot study was to investigate the acute effects during strength exercises and chronic effects of regular strength training on cfDNA concentrations over a period of four weeks in the following training groups: the conservation training group (CT), the high intensity-low repetition training group (HT) and the differential training group (DT). The CT started with a low intensity repetition exercise of 60% of their one repetition maximum (1RM) and the HT started with a traditional high intensity-low repetition exercise of 90% 1RM. The systematic indications for proceeding in the differential training group (DT) were variations of initial and/or final conditions of a movement, change of variables range, changing of a movement’s time course with respect to relative and absolute duration and rhythm [15]. These three general possibilities of variation can be applied to every joint to produce variations in angle, joint angular velocity and joint angular acceleration [15]. Positive results for the differential learning (DL) approach applied to strength training in the squat were shown by Hegen et al. [16]. The variability of motion should therefore eliminate the load reduction. In fact, this training style produces a maximum force load by the way of motion of the barbell [15,17].

Furthermore, we analysed the release of DNA-fragments sizes during acute exercise and at rest in the chronic exercise setting. Capillary blood plasma samples were taken from sixteen male subjects before every training session and on the first and the last training day repeatedly after every set. In addition, venous blood samples were taken 3 times a week before exercising to measure the CK levels. This pilot study was designed as a feasibility study testing the capability of cfDNA to react upon acute bouts of exercise as well as a regular strength exercise regime.

## Material and methods

### Ethical approval

All experimental procedures were approved by the Human Ethics Committee of Rhineland-Palatinate and conformed to the standards of the *Declaration of Helsinki of the World Medical Association*. All subjects were informed orally and in written form about the procedures and the aim of the study and gave written consent to participate.

### Subjects

A total of 16 male subjects who performed regular strength training for at least six months participated in a controlled study over a period of four weeks. Trained subjects were chosen in order to minimize the influence of coordinative learning processes in the early beginning of adaptation. Women were not included in order to minimize other hormonal influence [18]. Six subjects were assigned to CT and five subjects were assigned to HT and DT, respectively. HT applied standard maximum force workouts [17] while DT applied a setup of differential strength training and CT a conservation training [16]. After analyzing the pre-test result of their one repetition maximum (1RM) we assigned all sixteen subjects to either the CT, DT or HT group in order to parallelize the groups and minimize any side effects concerning their physical strength. The subjects assigned to the different groups did not differ in terms of their demographic characteristics (mean (± SD) age: 24.68 (2.27) years; height: 180 (8.03) cm; weight: 77.78 (8.03) kg; BMI: 24 (2.38)).

### Study design

Each participant received a training plan for three days a week consisting of two sets of eight strength exercises. Between each training session the subjects had to rest for 48 hours during the week and for 72 hours over the weekend. Experimental testing took place between 10:00 AM and 3:00 PM. Each training session lasted about 60 minutes and was supervised by a sport scientist and a medical student. The workout was performed at the same time each training day in order to avoid circadian differences in blood parameters. The subjects were requested to abstain from further physical exercise during the entire study period. None of the study participants were taking any relevant medication or had signs of infection. The subjects were assessed for physical activity via a standardized questionnaire.

Before starting the training intervention, every athlete performed a one repetition maximum (1RM) for each exercise. The 1RM method is a reliable and simple approach to determine the maximum force in a muscle or muscle group during a single exercise. This value was the base for the training recommendation in the three intervention groups [19,20]. All participants were randomly assigned to one group. Parallelization was done in balance with the results of the 1RM. Every single 1RM test result was aggregated, so the total amount of load that was moved by a subject could be defined.

The setup of the conservation training (CT) group was strength conservation and started with a low intensity repetition exercise of 60% 1RM with two sets and five repetitions each. The high intensity-low repetition training (HT) group started with a traditional high intensity-low repetition exercise of 90% 1RM. The intensities were chosen according to classical strength training recommendations [17]. The load was progressively increased by 2.5kg if the athlete completed one set with five repetitions. The workout of the differential training (DT) group also consisted of two sets and five repetitions each. According to classical strength training theory 90% 1RM should lead to increase of 1RM whereas with 60% 1RM mainly a muscular adaptation to the load is intended without increasing the 1RM. Hence, the goal was to increase the 1RM with lower intensity (60%) and higher movement variations during the execution of the exercise [15,16]. Subjects were only supposed to perform five repetitions, although they could have performed more concerning the intensity (load).

Basic exercises included two whole body exercises, deadlifts and squats. For specific muscle groups, training of arms, chest and back was applied (Table 1).

**Table 1:** Type of exercises with muscles targeted.

Muscle/ body parts	Whole body	Whole body	M. pectoralis major	M. biceps brachii	M. triceps brachii	M. latissimus dorsi	M. rectus abdominis	M. deltoideus (all parts)
Exercise	Deadlift	Squat	Bench press	Barbell curls	Cable push down	Flat bench pull	Crunches with extra load	Shoulder press (90°) with barbell

### Biological methods

#### Blood sampling and processing

To monitor the levels of cfDNA during one unit of exercise training, 20 μl of capillary blood was collected with a dipotassium-EDTA covered Microvette^®^ CB 300 (Sarstedt, Nümbrecht, Germany) from the fingertip of each subject before and after each exercise and at the beginning and the end of the training session. In addition, we collected blood samples from the fingertip before every single training session. Due to the strictly defined training session tardiness of the subjects led to 7 missing values during the entire study period (one missing value at study day 1,2 and 5 and four missing values at study day eight). To avoid sweat accumulations in the blood samples, the collection site was cleaned prior to blood collection. 20 ml of blood were taken from the antecubital vein before every training unit. 5.4 ml of the venous blood was sent to an external laboratory for the analysis of complete blood counts (e.g. erythrocytes, urea, mean corpuscular hemoglobin (MCH), hemoglobin (Hb), glutamate pyruvate transaminase (GPT)) and creatine kinase (CK). Capillary and venous blood samples were centrifuged immediately after collection at 4°C, 1600*g for 10 min. The plasma supernatants were high-speed centrifuged at 4°C, 16,000*g for 5 min to remove cellular debris. The samples were stored at -20°C for up to 4 months until qPCR measurement.

#### Quantitative real-time PCR reaction and conditions

cfDNA concentrations were quantified by analyzing unpurified plasma via qPCR with primers targeting the noncoding long interspersed nuclear element (LINE) L1PA2 family as described before [14]. Briefly, reactions were performed on a CFX384 Touch^TM^ Real-Time PCR detection system (Bio-Rad, München, Germany). The amplification consisted of an initial denaturation at 98°C for 2 min, followed by 35 cycles of melting at 94°C for 10 s, annealing at 64°C for 40 s and extension at 75°C for 10 s. The program Primer3 was used to design two primer sets for a 90-bp L1PA2 amplicon (L1PA2_90_) with 3,345 matches and a 222-bp L1PA2 amplicon (L1PA2_222_) with 3,134 matches in the human genome. For both amplicons the forward primer sequences was 5´-TGC CGC AAT AAA CAT ACG TG-3´, while the reverse primers sequences were 5´-GAC CCA GCC ATC CCA TTA C-3´ for L1PA290 and 5´-AAC AAC AGG TGC TGG AGA GG-3´ for L1PA2222 [14].

### Statistical analysis

The qPCR data was captured with the CFX Manager Software version 3.0 (Biorad, München, Germany). Microsoft® Excel 2007 was used for raw data collection. Statistical analysis was done using JMP 11 (SAS Institute Inc., Cary, NC, USA) and Microsoft^®^ Excel 2007 (Microsoft Corp., Redmond, WA, USA). CfDNA and CK values had to be log-normalized to achieve normal distribution as determined by Wilk-Shapiro W-test and equal variance as determined by the Levene’s test. Student’s T-testing was used for comparisons of normally distributed values. For non-normally distributed values we performed a median-test. CfDNA concentrations below the LOQ were excluded from statistical analysis [14]. For the analysis of acute effects, we computed a multi-factorial analysis of variance with log cfDNA concentrations as target variable, type of training (CT, HT and DT), number of exercise (1^st^ to 16^th^ repetition) and point in time for acute monitoring (1^st^ day or 12^th^ day) as influential factors. The chronic effects were also analysed in a multi-factorial analysis of variance with log cfDNA concentrations as target variable, type of training (CT, HT and DT), and point in time for chronic monitoring (study day 1, 2, 5, 8, 12) as influential factors. In the post-hoc analysis the significance level was adjusted to the number of comparisons by Bonferroni-Holm procedure. We considered p values of < 0.05 to be statistically significant. All data was presented as mean (± SD) or as de-logarithmized differences, therefore resembling fold-changes and their 95% confidence intervals (CI).

## Results

### Demographic characteristics and clinical parameters

Overall, sixteen male subjects participated in the study. The subjects trained 71.25 (21.37) minutes on average per week and had a training experience of 3.9 (2.7) years. During the training study all subjects completed 12 training sessions consisting of two sets of eight exercises, five repetitions each at different loads (60 and 90% 1RM). Demographic characteristics and clinical parameters of the subjects are presented in Table 2.

**Table 2:** Demographic characteristics and clinical parameters of study subjects at baseline.

	CT (n = 6)	HT (n = 5)	DT (n = 5)
**Demographic characteristics**			
Age (years)	25.16 (2.56)	25.0 (1.58)	23.8 (2.68)
Weight (kg)	74.98 (10.06)	81.32 (7.27)	77.6 (5.94)
Height (cm)	175.33 (8.77)	181.6 (5.12)	184.4 (7.7)
BMI	24.38 (2.75)	24.65 (2.02)	22.89 (2.56)
Strength training experience (years)	3.33 (3.10)	3.6 (1.74)	4.8 (2.63)
Strength training hours per week (minutes)	72.5 (23.18)	87 (11.22)	54 (12)
**Clinical parameters**			
CK (U/l) †	174.33 (63.49)	228 (161.0)	145.8 (56.94)
cfDNA L1PA2_90_ (ng/ml)†	6.75 (0.74)	10.48 (5.14)	6.65 (1.20)

† Median-test. The values are depicted as mean (± SD).

Abbreviations: BMI, body mass index; CK, creatine kinase; cfDNA, cell free DNA.CT, conservation training; HT, high intensity-low repetition training; DT, differential training.

There were no significant differences in baseline cfDNA values between the three training groups (mean (±SD) CT 6.75 (0.74) ng/ml), HT 10.48 (5.14) ng/ml and DT 6.65 (1.20) ng/ml)

### Association between cfDNA concentrations and strength exercise after a single strength exercise training session

**Fig 1:**
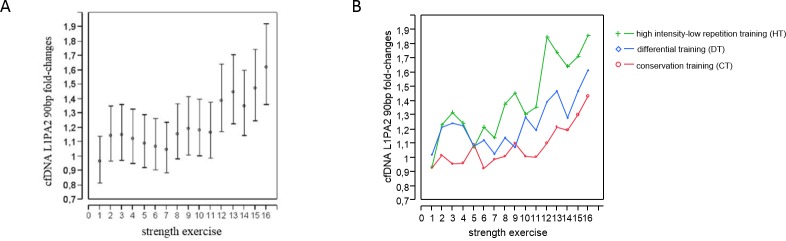
Significant increases of cfDNA levels after the 12^th^ exercise compared to baseline levels. (A) Fold changes of cfDNA concentrations after each strength exercise (1–16) compared to baseline levels across all types of training (CT, HT and DT) and at both monitoring points in time (1^st^ and 12^th^ day). Means (circles) and 95% CIs are presented. (B) Fold changes of cfDNA concentrations after each strength exercise (1–16) compared to baseline levels shown separately for CT, HT and DT group.

To analyse the effects of a single training session on cfDNA levels we determined the fold changes of cfDNA concentrations after each exercise compared to baseline levels (Fig 1).

In an multifactorial analysis of variance across all types of training (CT, HT and DT) and at both monitoring points in time (1^st^ and 12^th^ day) there was a significant increase of cfDNA after the 12^th^ exercise compared to baseline (95% CI 1.17–1.64; p = 0.002). Thereafter, cfDNA concentrations increased significantly and steadily compared to baseline concentrations (13^th^ exercise: 95% CI 1.22–1.71; p<0.001, 14^th^ exercise: 95% CI 1.14–1.6; p = 0.005, 15^th^ exercise: 95% CI 1.25–1.74; p<0.001, 16^th^ exercise: 1.62-fold, 95% CI 1.36–1.93; p<0.001). Across all points in time and uncorrected against multiple comparisons cfDNA concentrations were 1.77-fold higher in HT (mean (±SD) before first exercise: 12.23 (6.29) ng/ml, after last exercise 17.73 (11.24) ng/ml) compared to CT (mean (±SD) before first exercise: 6.79 (1.28) ng/ml, after last exercise 10.05 (2.89) ng/ml) (95% CI 1.21–2.58; p = 0.01). In addition we observed only 1.26-fold higher concentrations for DT (mean (±SD) before first exercise: 7.32 (2.89) ng/ml, after last exercise 12.7 (3.02) ng/ml) in comparison to CT (mean (±SD) before first exercise: 6.79 (1.28) ng/ml, after last exercise 10.05 (2.89) ng/ml) (95% CI 0.85–1.81). However, as shown in Fig 1B, this did not lead to any significant differences between the groups for the changes in cfDNA concentrations compared to baseline. There was no significant difference between the two monitoring points in time (1^st^ and 12^th^ day) with both showing similar response to acute exercise.

### Effects of chronic strength training on cfDNA levels and blood parameters

**Fig 2:**
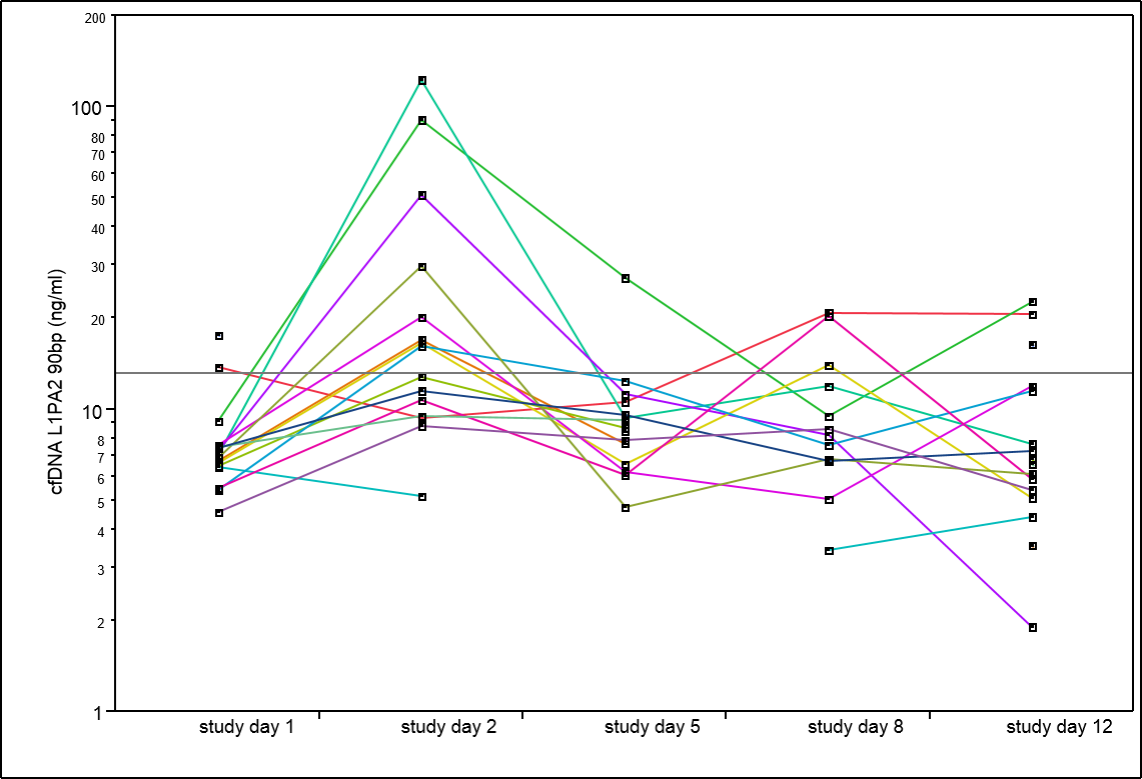
Significant increases of cfDNA levels at study day two compared to baseline levels. Time course of total cfDNA concentrations during the four-week study in all training groups before the exercise sessions. Missing values lead to interrupted lines (see methods).

To analyse the effects of chronic strength training on the concentration of cfDNA in blood plasma we collected capillary blood samples before the beginning of the session at each training day before exercising (Fig 2).

Concerning the chronic effects of strength exercise, we observed significant differences comparing the baseline cfDNA levels before intervention (study day 1) and at study days 2, 5, 8 and 12 (**χ**^**2**^ = 20.09, n = 4, p = 0.002). Total cfDNA base line levels increased from 8.31±2.84 ng/ml at the first study day to 28.82 ±32.906 ng/ml (3.47-fold). CfDNA concentrations at study day two were significantly increased compared to baseline levels (**χ** ^**2**^ = 16.50, n = 1, p<0.001) and also significantly higher than on study day 5 (mean (±SD) 9.73 (5.08) ng/ml, 1.23-fold) (**χ**^**2**^ = 7.01, n = 1, p = 0.02). Type of training did not show a significant association with the changes in cfDNA values.

**Fig 3:**
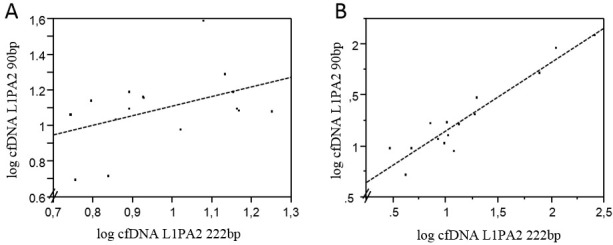
Increases in cfDNA at rest, due to previous training sessions mostly involve increases in polynucleosomal DNA. Linear correlations of total nucleosomal cfDNA (cfDNA ≥ 90bp) with polynucleosomal cfDNA (cfDNA ≥ 222bp) following a single training session at study day one (A) and at study day two (B). While a highly significant correlation was found at study day two (r = 0.95; p < 0.0001), no significant correlations were found at study day one (as shown in A).

Following the first single training session on study day 1, cfDNA increases (total nucleosomal cfDNA, cfDNA fragments ≥ 90bp) are higher and not correlated with increases in polynucleosomal cfDNA (cfDNA fragments ≥ 222bp; Fig 3A). Concerning the chronic effects amongst all study days only day 2 showed a highly significant correlation of polynucleosomal with total nucleosomal cfDNA concentrations (r = 0.95; p < 0.0001, Fig 3B). In fact, levels of polynucleosomal DNA are similar to those of all total nucleosomal DNA (total nucleosomal cfDNA, cfDNA fragments ≥ 90bp) indicating that increases in cfDNA at rest, due to previous training sessions mostly involve increases in polynucleosomal DNA.

Concerning the blood parameters, we detected significant increases of hemoglobin (Hb) and mean corpuscular hemoglobin (MCH) after study day 5 compared to baseline values (Hb: 95% CI 1.00–1.04; p = 0.02, fold changes 1.02; MCH: 95% CI 1.01–1.02; p<0.001, fold changes: 1.01). In addition, we could measure a significant increase in urea after study day 5 compared to baseline values (95% CI 1.08–1.35; p<0.001, fold changes: 1.21).

### Evaluation of cfDNA and creatine kinase concentrations during chronic strength exercise

**Fig 4:**
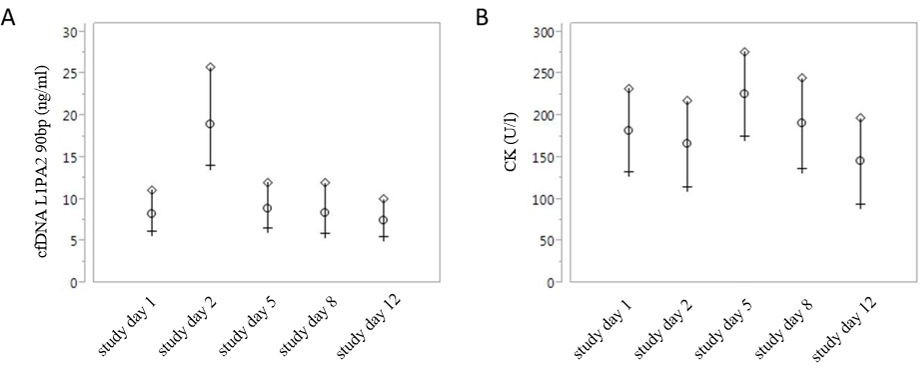
Training procedures only causes alterations in cfDNA and not in CK levels. Kinetics of cfDNA (A) and CK concentrations (B) during the 4 week intervention. Mean (circles) and 95% upper (diamonds) and lower (crosses) values are presented.

To compare the kinetics of cfDNA and creatine kinase (CK) concentrations, we collected venous blood samples before every training day (Fig 4).

As shown before, we detected a significant increase of capillary cfDNA values two days after the first training session. Compared to the cfDNA levels, we could not detect a simultaneous increase of CK concentrations at study day 2 (study day 1: 182.18 (± 101.42) U/l, study day 2: 165.13 (± 60.13) U/l. Moreover, we detected no overall significant changes of CK concentrations during the entire intervention period (p = 0.28).

## Discussion

Up to date, only creatine kinase (CK) is a marker for muscle cell damage with limited potential as marker for training load in strength training. Here we wanted to investigate the acute effects during strength exercises and chronic effects of regular strength training on cfDNA concentrations over a period of four weeks in three training groups and to analyse the sizes of DNA fragments released after acute and chronic exercise settings.

Therefore, we analysed the effects of short-term resistance training on cfDNA levels. We detected a significant increase of cfDNA levels after the 12^th^ exercise compared to baseline levels. Our results of only moderate increases of cfDNA levels (1.62 fold) were lower than the increases of cfDNA levels measured by Atamaniuk *et al*. after a single bout of strength exercise (3.3 fold). The authors concluded that increases of cfDNA concentrations might represent an index of muscle tissue damage, cellular necrosis and apoptosis following an acute exercise-induced (muscle) injury. Focusing on strength exercise, Atamaniuk *et al*. chose a higher training intensity and volume than our study protocol, where only five volunteers in HT trained with 90% 1RM. This could be the reason why they detected a higher cfDNA increase compared to our study. In addition, our high intensity-low repetition training group (HT) showed higher cfDNA values during a single unit of strength training than the conservation training group (exercising with 60% 1RM). However, it is described that the mobilization and activation of leukocytes, the generation of oxidative stress and the release of acute phase proteins are dependent on duration and intensity of exercise exposure [21]. To our knowledge, we monitored in our study for the first time cfDNA levels over the course of a training program, showing steady increases from set to set. In line with our observations during acute running exercises the type of cfDNA released was rapid and the main proportion of DNA was mononucleosomal in size. In healthy patients, cfDNA fractions are mostly derived from apoptosis of various blood cells that generate small fragments (polynucleosomal) of cell-free DNA, whereas the cell-free circulating DNA of cancer patients represents a mix of apoptosis, necrosis, autophagy, or mitotic catastrophe. Necrosis produces relatively long fragments of DNA (mononucleosomal), about 10,000bp in length, while in apoptosis, the activation of endogenous endonucleases lead to the cleavage of chromatin DNA into internucleosomal fragments. In a follow up study cfDNA levels should be monitored using a study design with higher training intensities and training volume to analyse if there are correlations between the training intensity and volume and the release of mononucleosomal DNA fragment sizes. In addition, post-performance values should be collected to analyse differences between acute and chronic effects of strength exercise training on cfDNA levels.

The chronic effects of strength exercise on cfDNA and CK levels indicated that the training procedures did not cause any alterations in CK whereas cfDNA levels increased significantly at study day 2 compared to baseline levels and compared to study day 5. Most of the studies, which analysed the effect of exercise on cfDNA levels, detected a decrease of cfDNA concentrations to baseline levels two hours after exercise [4,9]. Only two studies documented elevated cfDNA levels two hours after an ultra-endurance exercise [9] and 96 hours after overtraining [13]. The increase of cfDNA levels on training day 2 could be attributed to a local inflammatory reaction which happens through direct mechanical lesions of the muscle sarcomere [22] and indirect formation of oxygen free radicals [23–25]. Within hours, neutrophils migrate into the injured skeletal muscle and remain there up to 24 hours. Together with macrophages they degrade the damaged muscle tissue resulting in the formation of ROS [26]. Atamaniuk *et al*. suspected that cfDNA represents a marker of muscle damage, apoptosis or necrosis [9]. In this study we can now show for the first time that late increases in cfDNA that occur more than 24 hours after the last strength training session (here on study day two) are mostly polynucleosomal cfDNA-fragments in nature. This lower level of degradation of the cfDNA could resemble a release of less degraded DNA due to cellular damage. In addition, we detected only on study day 2 elevated cfDNA levels at rest, with a highly significant correlation of polynucleosomal with all nucleosomal cfDNA concentrations. Therefore, levels of polynucleosomal DNA are similar to those of all total nucleosomal DNA indicating that increases in resting cfDNA due to a previous training session mostly involve increases in polynucleosomal DNA. In a follow up study, the influence of high training intensities and volume on polynucleosomal DNA fragments should be analysed and compared to CK values, to figure out, which marker can better reflect the total exhaustion of the muscles. However, the origin of the late release of cfDNA remains to be demonstrated. It was shown that chronic excessive resistance exercise leads to elevated levels of cfDNA in proportion to training load, suggests that cfDNA concentrations can be used as a marker for overtraining-induced inflammation [13]. Since the authors detected elevated levels of CK during their study period they concluded that the presence of cfDNA after strength training is an indication for muscle cell damage and chronic exercise-induced inflammation [13]. Even if we detected elevated cfDNA levels 48 hours after exercise, we did not perform such high intensity training as described by Fatouros *et al*. [13]. In addition, we also detected elevated cfDNA levels at study day two in our control group, who performed at 60% of their 1RM. Therefore, we do not expect that the increased cfDNA levels could be due to overtraining-induced inflammation. Furthermore, we did not find any changes in CK levels and assume that there are no relevant muscle cell damages caused by strength exercises in our study. Therefore, further studies will be needed to demonstrate the physiological meaning of increases in cfDNA at rest following particular training sessions. The decreases in hemoglobin and mean corpuscular hemoglobin and the increases in urea after study day 5 compared to baseline values could indicate late effects of exercise on blood parameters. It was already shown that blood parameters like urea, lactate or ammonia are typical substrates for measuring e.g. overtraining [27].

Taken together, we found a different response due to a single bout of strength exercise resembling a rapid type and inflammation related kind of cfDNA release. Our results suggest that during strength exercise short-fragmented cfDNA is released, reflecting a fast aseptic inflammatory response, while elevation of longer fragments at baseline on study day two seemed to reflect a mild cellular damage due to a novel training regime. Unfortunately there was no convincing difference and no clear dependence on resistance training protocol for the development by analyzing the fold-change development of the cfDNA for the three groups separately. However, we hope that clarifying the aspects of the quality of the released cfDNA in the acute setting versus the follow-up increases in the days thereafter will be helpful to design future studies on cfDNA as an exercise marker.

## Supporting information

S1 File List of the complete data set. (XLSX)
